# Genetic Association Analysis of *NOS3* and Methamphetamine-Induced Psychosis Among Japanese

**DOI:** 10.2174/157015911795017119

**Published:** 2011-03

**Authors:** T Okochi, T Kishi, M Ikeda, T Kitajima, Y Kinoshita, K Kawashima, T Okumura, T Tsunoka, Y Fukuo, T Inada, M Yamada, N Uchimura, M Iyo, I Sora, N Ozaki, H Ujike, N Iwata

**Affiliations:** 1 Department of Psychiatry, Fujita Health University School of Medicine, Toyoake, Japan; 2 Department of Psychiatry, Teikyo University Ichihara Hospital, Ichihara, Japan; 3 Department of Psychogeriatrics, National Institute of Mental Health, National Center of Neurology and Psychiatry, Kodaira, Japan; 4 Department of Neuropsychiatry, Kurume University Graduate School of Medicine, Kurume, Japan; 5 Department of Psychiatry, Chiba University Graduate School of Medicine, Chiba, Japan; 6 Department of Psychobiology, Tohoku University Graduate School of Medicine, Sendai, Japan; 7 Department of Psychiatry and Psychobiology, Nagoya University Graduate School of Medicine, Nagoya, Japan; 8 Department of Neuropsychiatry, Okayama University Graduate School of Medicine, Dentistry and Pharmaceutical Science, Okayama, Japan; 9 Japanese Genetics Initiative for Drug Abuse (JGIDA)

**Keywords:** Methamphetamine-induced psychosis, endothelial nitric oxide synthase (*NOS3*), gene-based case-control association study.

## Abstract

Endothelial nitric oxide synthase (*NOS3*) is one of the enzymes influencing nitric oxide (NO) function in the human brain. NO is a gaseous neurotransmitter that is involved in a variety of mechanisms in the central nervous system, such as N-methyl-D-aspartate receptor activation and oxidative stress. The evidence from animal pharmacological studies and postmortem studies supports an association between NO and psychotic disorders. Methamphetamine (METH) use disorder is a known psychotic disorder, and we therefore conducted a gene-based case-control study between tagging single nucleotide polymorphisms (SNPs) (rs2070744, rs1799983) in *NOS3* and METH-induced psychosis in Japanese subjects (183 with METH-induced psychosis and 267 controls). Written informed consent was obtained from each subject. No significant association was found between any tagging SNP in *NOS3* and METH-induced psychosis in the allele/genotype-wise or haplotype-wise analyses. In conclusion, we suggest that *NOS3* might not contribute to the risk of METH-induced psychosis in the Japanese population.

## 1 INTRODUCTION

Methamphetamine (METH) is an illegal drug used widely in the world, known to cause psychiatric disorder. METH releases dopamine in the central nervous system (CNS) [1]. Moreover, excess dopamine in the CNS is thought to cause psychotic symptoms such as hallucinations and delusions [2]. The symptomatologic character of METH-induced psychosis is similar to that of schizophrenia. A recent study reported METH may cause selective increase of Nitric oxide (NO) in the striatum, leading to dopaminergic neurotoxicity [3].

NO is a gaseous neurotransmitter involved in a variety of mechanisms in the CNS and the vascular system. This molecular signaling has a role in regulating other neurotransmitters, such as dopamine and serotonin that is involved in neuronal dysfunction in schizophrenia and mood disorder [4, 5]. Pharmacological studies in animal models reported an association between NO and behavioral abnormality caused by phencyclidine [6-8].

NO is synthesized from L-arginine by nitric oxide synthase. One of the enzymes influencing NO function in the human brain is endothelial nitric oxide synthase (*NOS3*).* NOS3* is located on chromosome 7q35-36, including 21 exons and spanning 24.33kb. Two functional SNPs (rs2070744, rs1799983) in *NOS3* have been reported and genetic association studies on them have been performed [9, 10]. Much attention has been focused on associations between these functional SNPs in *NOS3* and cardiovascular disease. It may be that *NOS3* controls brain blood flow, producing NO to regulate vascular tone. Additionally, *NOS3* knockout mouse showed a low rate of neural stem cell proliferation, and responsiveness in the learned helplessness paradigm, a promising animal model of depression [11]. However, there are few genetic association studies between *NOS3* and psychotic disorders [12]. Based on this evidence, we suspected that *NOS3* might be related to the pathophysiology of METH-induced psychosis, and conducted a genetic association analysis of six tagging SNPs, including two functional SNPs, in *NOS3* and METH-induced psychosis in the Japanese population.

## 2 MATERIALS AND METHODS

### 2.1 Subjects

The subjects in the association analysis were 183 patients (all patients were diagnosed as having METH-induced psychosis; 151 males and 32 females: mean age ± SD 36.7±11.6 years) and 267 healthy controls (217 males and 50 females: mean age ± SD 35.5 ±14.4 years). There was no significant association between the age of the healthy controls and that of the patients (Table **1**). All subjects were unrelated to each other, ethnically Japanese, and lived in Japan. Among the subjects with METH use disorder, all subjects had a comorbid diagnosis of METH-induced psychosis. One hundred forty-nine subjects with METH use disorder abused or had dependence on drugs other than METH. Cannabinoids were the most frequently abused drugs (31.4%), followed by cocaine (9.09%), LSD (9.09%), opioids (7.69%), and hypnotics (7.69%). Subjects with METH use disorder were excluded if they had a clinical diagnosis of psychotic disorder, mood disorder, anxiety disorder, or eating disorder. The patients were diagnosed according to DSM-IV or ICD-10 criteria with consensus of at least two experienced psychiatrists on the basis of unstructured interviews and a review of medical records. All healthy controls were also psychiatrically screened through unstructured interviews, and those with past individual or family history of drug dependence or axis 1 disorders such as psychotic or mood disorders were excluded. After describing the study, written informed consent was obtained from each subject. This study was approved by the Ethics Committee at Fujita Health University and each participating institute of the Japanese Genetics Initiative for Drug Abuse (JGIDA).

### 2.2 SNP Selection and Genotyping

We first consulted the HapMap database (release#21a, Jan 2007 www.hapmap.org, population: Japanese Tokyo: minor allele frequencies (MAFs) of more than 0.05) and found 32 SNPs covering *NOS3*. Then 5 ‘tagging SNPs’ were selected with the criterion of an *r2* threshold greater than 0.8 in ‘pair-wise tagging only’ mode using the ‘Tagger’ program (Paul de Bakker, http://www/broad.mit.edu/mpg/tagger), using the HAPLOVIEW software. We selected 6 SNPs that included functional polymorphisms (rs1800779, rs2070744, rs1799983, rs3918188, rs743507, rs7830) in *NOS3*.

We used TaqMan genotyping assays (Applied Biosystems) for all SNPs. 

### 2.3 Statistical Analysis

Genotype deviation from the Hardy-Weinberg equilibrium (HWE) was evaluated with the chi-square test (SAS/Genetics, release 8.2, SAS Japan INC, Tokyo, Japan). Marker-trait association was also evaluated with the chi-square test in allele- and genotype-wise analyses. Haplotype frequencies were estimated in a two- to four-marker sliding window fashion and log likelihood ratio tests were performed for global P-values with COCAPHASE program version 3.0.6 [13]. In these haplotype-wise analyses, rare haplotypes (less than 0.05) of either cases or controls were excluded from the association analysis. Power calculation was performed using a statistical program prepared by the Genetic Power Calculator (http://pngu.mgh.harv-ard.edu/~purecell//gpc/) [14]. The level of significance for all statistical tests was 0.05.

## 3 RESULTS

Genotype frequencies of subjects and controls did not deviate significantly from HWE. No significant association was found between *NOS3* and METH-induced psychosis in the allele/genotype-wise analysis (Table **2**) or in the haplotype analysis (Table **3**).

In a power analysis, we obtained more than 80% power for the detection of association when we set the genotype relative risk at 1.81-2.31, under a multiplicative model of inheritance.

## 4 DISCUSSION

In this study, we performed a genetic association study based on LD between *NOS3* and METH-induced psychosis. However, no association was found between *NOS3* and METH-induced psychosis in these Japanese subjects in allele/genotype-wise and haplotype-wise analysis.

In a recent study using knockout mice, Reif *et al.* looked for an association between *NOS3* and mood disorders. They suggested that a haplotype including two functional SNPs (rs2070744, rs1799983) in *NOS3* may influence the susceptibility of bipolar disorder [12]. Additionally, Kawohl *et al.* found an association between rs1799983 and lower responsiveness in the loudness dependence of auditory evoked potentials (LDAEP), which is a functional marker of serotonergic transmission [15]. Several pieces of evidence have suggested *NOS3* has an important role in the serotonin system in the human brain. Therefore, it will be necessary to replicate these associations with the same phenotype and others using more samples.

A few points of caution must be mentioned with regard to our findings. (3) It is important to evaluate associations between METH use disorder with and without psychosis. However, only a small number of subjects had no psychosis, and so we did not evaluate this association in order to avoid type I error from a small sample size. The small sample size was due to the limitations of sample collection, since we used cases of METH use disorder in psychiatric hospitals. (13*-) We did not include a mutation scan for rare variants. Because rare variants with functional effects in *NOS3* have the possibility of influencing susceptibility of METH-induced psychosis, further investigations including mutation scan using large samples will be required.

In conclusion, our results suggest that *NOS3* does not play a major role in METH-induced psychosis in the Japanese population. However, the number of METH patients used in this study was small. It will be necessary to validate or replicate our association in other, larger population samples.

## Figures and Tables

**Table 1:** Characteristics of METH-Induce Psychosis and CON Subjects

	METH-Induced Psychosis	CON	*P*-value
N	183	267	
male	151	217	
female	32	50	0.737
Age means ± SD	36.704 ± 11.6	35.527 ± 14.44	0.309

**Table 2:** Association Analysis between NOS3 and METH-Induced Psychosis

SNP ID	Phenotype^a^	MAF^b^	N	Genotype Distribution^c^			*P*- Value		
				M/M	M/m	m/m	HWE^d^	Genotype	Allele
rs1800779	METH-induced psychosis	0.117	183	140	43	0	0.0717	0.139	0.248
	CON	0.0936	267	219	46	2	0.805		
rs2070744	METH-induced psychosis	0.120	183	140	42	1	0.250	0.357	0.168
	CON	0.0918	267	219	47	1	0.359		
rs1799983	METH-induced psychosis	0.0847	183	152	31	0	0.210	0.500	0.787
	CON	0.0899	267	221	44	2	0.906		
rs3918188	METH-induced psychosis	0.246	183	107	62	14	0.242	0.286	0.424
	CON	0.270	267	140	110	17	0.452		
rs743507	METH-induced psychosis	0.156	183	130	49	4	0.805	0.848	0.609
	CON	0.169	267	183	78	6	0.489		
rs7830	METH-induced psychosis	0.484	183	49	91	43	0.952	0.496	0.399
	CON	0.455	267	85	121	61	0.158		

a METH:methamphetamine CON:control

b MAF: minor allele frequency

c M: major allele, m: minor allele

d Hardy-Weinberg equilibrium

**Table 3:** Results of Haplotype Analysis between *NOS3* and METH-Induced Psychosis

Global *P*-value			
SNP ID	2 SNP	3 SNP	4 SNP
rs1800779			
	0.465		
rs2070744		0.659	
	0.656		0.653
rs1799983		0.723	
	0.548		0.733
rs3918188		0.649	
	0.408		0.515
rs743507		0.410	
	0.622		
rs7830			
